# Ebola Virus Maintenance: If Not (Only) Bats, What Else?

**DOI:** 10.3390/v10100549

**Published:** 2018-10-09

**Authors:** Alexandre Caron, Mathieu Bourgarel, Julien Cappelle, Florian Liégeois, Hélène M. De Nys, François Roger

**Affiliations:** 1 CIRAD, UMR ASTRE, RP-PCP, Harare, Zimbabwe; mathieu.bourgarel@cirad.fr; 2 ASTRE, Uni. Montpellier, CIRAD, INRA, 34398 Montpellier, France; julien.cappelle@cirad.fr (J.C.); helene.de_nys@cirad.fr (H.M.D.N.); francois.roger@cirad.fr (F.R.); 3 Faculdade de Veterinaria, Universidade Eduardo Mondlane, Maputo 01009, Mozambique; 4 UMR EPIA, INRA, VetAgro Sup, Univ Lyon, F-69280 Marcy-l’étoile, France; 5 CIRAD, UMR ASTRE, 34398 Montpellier, France; 6 UMR 224, MIVEGEC, IRD/CNRS/Uni. Montpellier, 34394 Montpellier, France; florian.liegeois@ird.fr; 7 UMR 233 TransVIHMI, IRD/Uni. Montpellier/INSERM, 34394 Montpellier, France

**Keywords:** Africa, bat, community ecology, ebola virus, filovirus, maintenance host, transmission pathways

## Abstract

The maintenance mechanisms of ebolaviruses in African forest ecosystems are still unknown, but indirect evidences point at the involvement of some bat species. Despite intense research, the main bat-maintenance hypothesis has not been confirmed yet. The alternative hypotheses of a non-bat maintenance host or a maintenance community including, or not, several bat and other species, deserves more investigation. However, African forest ecosystems host a large biodiversity and abound in potential maintenance hosts. How does one puzzle out? Since recent studies have revealed that several bat species have been exposed to ebolaviruses, the common denominator to these hypotheses is that within the epidemiological cycle, some bats species must be exposed to the viruses and infected by these potential alternative hosts. Under this constraint, and given the peculiar ecology of bats (roosting behaviour, habitat utilisation, and flight mode), we review the hosts and transmission pathways that can lead to bat exposure and infection to ebolaviruses. In contrast to the capacity of bats to transmit ebolaviruses and other pathogens to many hosts, our results indicate that only a limited number of hosts and pathways can lead to the transmission of ebolaviruses to bats, and that the alternative maintenance host, if it exists, must be amongst them. A list of these pathways is provided, along with protocols to prioritise and investigate these alternative hypotheses. In conclusion, taking into account the ecology of bats and their known involvement in ebolaviruses ecology drastically reduces the list of potential alternative maintenance hosts for ebolaviruses. Understanding the natural history of ebolaviruses is a health priority, and investigating these alternative hypotheses could complete the current effort focused on the role of bats.

## 1. Introduction

Ebolaviruses (EBVs), according to Kuhn et al. classification [1]) are single-strand RNA filoviruses that can induce a high mortality in some hosts, including apes and humans [2,3]. The different ebolaviruses have caused localised but dramatic human outbreaks, mainly in Central Africa, in the last 40 years. The recent West African outbreak in 2013–2016 gave an outline of the pandemic potential of these pathogens [4,5].

EBVs are zoonotic pathogens. Several EBV species in Africa have spilled over into human populations several times from animals to humans, with primary cases (confirmed or suspected) occurring close or within forest habitats and often after close contacts (e.g., hunting, transport, eating) between humans and forest wild species (including duikers, monkeys, and great apes) [3,6,7,8,9,10,11,12,13,14,15]. The disease ecology of EBV is, therefore, multi-hosts and deeply entrenched at human/wildlife interfaces within forest ecosystems. There is a need to understand what constitutes the maintenance of EBV (that could differ from one EBV species to another), namely, a maintenance host or a maintenance community, i.e., several species in interaction within specific forest ecosystems. Understanding how this maintenance system works could help in predicting and preventing future outbreaks. Here, we briefly present current and discuss alternative hypotheses in order to provide insight into different avenues of investigation. We will focus on the disease ecology of the Zaire ebolavirus (EBOV) for simplicity, but most of the following hypotheses apply to the other African EBVs.

## 2. Current Hypotheses for the Maintenance of EBOV

**Figure 1:**
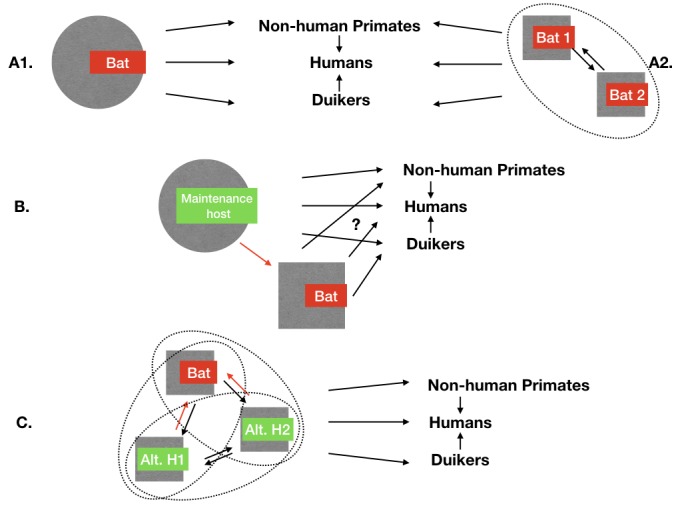
Potential maintenance mechanisms of ebolaviruses in wildlife, according to current knowledge. Circles (plain or dotted) indicate a maintenance function play by the host(s); arrows represent infectious transmission pathways between hosts. Humans, non-human primates, and duikers are examples of known non-maintenance hosts, exposed occasionally to ebolavirus directly or indirectly through the main maintenance host. (**A1**) Main maintenance hypothesis: there is one bat species maintaining each ebolavirus alone. Currently this is logically the most investigated hypothesis given the available data, and represents the maintenance mechanism for another filovirus, the Marburg virus, as currently understood. (**A2**) Several bat species are needed to create a maintenance community for Zaire ebolavirus (EBOV); each bat species cannot complete EBOV maintenance alone, as it requires interactions with the other species. (**B**) Alternate non-bat maintenance host hypothesis: if it exists, it is known that it can transmit ebolaviruses to some bat species. In this article, we review the potential hosts and associated transmission pathways that link this host to bat species (red arrow). (**C**) The maintenance community hypothesis, in which several hosts are needed to maintain ebolaviruses (ellipses represent different scenarios of community maintenance). This could be one or more alternative hosts involving possibly bat species. By definition, if such an alternative host exists, there are infectious transmission pathways from this host towards bats that are reviewed here (red arrows).

Disentangling the complexity of maintenance hosts or communities in multi-host systems at the wildlife/livestock/human interface is a difficult task [16,17,18]. The maintenance of EBV in equatorial forests is yet to be understood. Some mammal species played a major role in triggering human outbreaks: apes such as chimpanzees (*Pan troglodytes troglodytes* and *P. t. verus*) and western lowland gorillas (*Gorilla gorilla gorilla*) were at the origin of several human outbreaks [10,11,12], but have been found to be highly susceptible to EBV with potential drastic impact for their populations [12,19]. EBOV PCR positive duiker carcasses (*Cephalophus* sp.) have also been found [20]. One would not expect such a high mortality (relative to their population density) of EBOV in maintenance hosts. However, these data indicate their possible involvement in the transmission function of EBOV, bridging the maintenance host with human populations during a spillover event [18] (Figure 1). The EBOV susceptibility and exposure (tested by virology, serology and/or PCR) of many other potential forest hosts, including invertebrates, birds, bats, monkeys, rodents, and other small mammals, have been tested in the field or experimentally with an interestingly large amount of negative results (e.g., [12,21,22,23,24,25,26]). A few monkey and bat individuals serologically positive to EBV antigen represent the only exceptions [12].

Today, African bats are considered by many as the best candidates for acting as maintenance hosts for EBOV. Partial vRNA was sequenced from living specimens of three different bat species in Central Africa [23], and antibodies against ebolavirus antigen have been detected in 9 bat species (8 frugivorous and 1 insectivorous) [3,23,27,28,29,30]. Recently, a new ebolavirus species with an unknown pathogenic risk has also been isolated from two insectivorous bat species roosting inside a house [31]. Moreover, Swanepoel et al. showed that EBOV replicated in three species of experimentally infected bats (*Tadarida condylura*, *Tadarida pumila*, and *Epomophorus wahlbergi*), including virus isolated from faeces 21 days after experimental infection [22]. In addition, some bat species have been shown to act as maintenance hosts for multiple RNA viruses, including filoviruses (e.g., [32,33,34]). However, to date, no EBOV replicative strain has been isolated from healthy wild bats despite thousands of individuals tested [14,23,24,25,28,34,35]. Given the current knowledge, the main hypotheses for EBOV maintenance are a single bat species as *Rousettus aegyptiacus* is considered the maintenance host for Marburg virus (Figure 1A1); or a network of interacting bat species creating a maintenance community for EBOV (Figure 1A2).

The bat system is complex. First, for its diversity: globally, they represent over 20% of the mammal diversity, forming the second largest mammalian order after rodents, and Africa hosts 317 known living species, 25% of the global bat diversity [36]. Secondly, bats have exceptional lifestyles that have already been reviewed, especially in relation to their role in disease ecology [33,37,38,39,40,41,42,43]. They are unique mammal species regrouping such peculiar life history traits as their aerial life mode, their longevity, their gregarious and migration patterns, as well as their immune system.

Proving that a bat species maintains EBOV (e.g., [44,45]), or that interconnected populations of different bat species create the cradle for EBOV maintenance in a specific ecosystem, is a difficult task. Finding a live virus in a healthy bat specimen would constitute a great step in proving that this particular species is part or the totality of the EBOV maintenance. However, this finding would also trigger new questions: does this species act alone to maintain EBOV, or do other sympatric bat species’ populations create a maintenance community for EBOV? Is this EBOV maintenance system unique or ecosystem specific? Additionally, are other non-bat species involved in the maintenance? The road to identifying the maintenance host(s) of EBOV is still long.

The gaps in knowledge concerning the maintenance of EBOV and other EBV are therefore still significant. Available data indicates a systematic but weak signal in some bat species, a pattern in line with the main bat maintenance hypotheses, but not excluding as well alternative hypotheses as presented in Figure 1B,C. If those alternative scenarios do not necessarily agree with the Occam’s razor principle, they still cannot be ignored by the scientific community. African forest ecosystems host a high diversity of organisms relative to other ecosystems, and provide a rich pool of candidate species for playing a role in EBOV maintenance. EBOV specialists agree in calling for more integrated efforts across scientific fields, notably epidemiology, ecology, molecular biology, remote sensing modelling, and social sciences to test new hypotheses [39]. We provide, here, an ecological perspective on the EBOV multi-host system to provide a hypothesis-driven framework for future work. There is still a possibility that bats are not part of or that non-bat species are involved in the EBOV maintenance system and alternative scenarios should be considered and explored (Figure 1) [46]. These scenarios should be investigated, when possible, alongside bat-centred protocols, to confirm or invalidate the case for bats as EBOV maintenance hosts.

## 3. What If Bats Are Not the (Only) Maintenance Hosts for EBOV?

When a probability *P* is difficult or impossible to estimate, it is sometimes easier to estimate its inverse probability (*1-P*), the probability that it does not happen. It would be tedious to quantitatively estimate probabilities in the case of ebolavirus maintenance given the current lack of information, but trying to define the components of this probability could help. Hence, instead of proving that bats are the maintenance host for EBOV, what if we consider that “*bats are not the (only) maintenance host for EBOV*”?

Here, we consider the scenario presented in Figure 1B,C, namely, that bats are not the maintenance host for EBOV or that bat species are involved with alternative host(s) in the EBOV maintenance community. Current data and knowledge support both scenarios. Some bats are sometimes in contact with the virus and experience waves of exposure during outbreaks [27]. Once infected, bats could either be dead-end hosts, as some experimental studies suggest that some bat species cannot excrete the virus [47]); or they could transmit viruses to other hosts, such as primates including humans [6,48,49] as a bridge host, linking the maintenance host with humans. This means based on the definition of a bridge host [18], that these bats must have been in contact, at some point in the epidemiological cycle, with the maintenance host (or another bridge host) to get the EBOV infection. Here, “contact” means infectious contact, and can be direct (e.g., physical) or indirect (e.g., through the environment). The search for alternative maintenance hosts for EBOV should, therefore, concentrate on hosts that can transmit the virus to bats. In other words, any host that could not transmit the virus to bats would be ineligible to be a maintenance host for EBOV. This holds for any host found exposed to EBOV (e.g., some duiker sp.) but the focus on bats is justified in the following section.

The ecology of most African bat species is largely unknown. It can still be summarised as follows: roosting in trees (hanging or in holes) or caves, flying, eating insects while flying (insectivorous bats)/eating fruits in trees (fruit bat), flying back and roosting in trees or caves; with biannual long-range migration or nomadic movements for some species [50]. A single bat can cover a large variety of habitats and even regions for those migrating. Therefore, the transmission pathways from bats to other animals through urine, saliva, birthing fluids, and placental material and/or guano could be important (see review on Ebola isolated from body tissues and fluids [51]). Predation is also a less known but potential transmission pathway from bats to predators [48,52]. The range of potential species at risk of infection from bats is thus large [53]. However, the range of potential transmission pathways available for the maintenance or bridge host (under scenario B and C in Figure 1) to infect bats seems to be much more limited. For example, bats seldom use the ground floor: transmission routes requiring direct contact or environmental transmission on the ground do not expose bats. In other terms, direct contacts with strictly ground-dwelling animals would be very unlikely. Four habitat types structure the various transmission pathways from the alternative host to bats (and each bat species will frequent only a fraction of these habitats: (i) open air while flying, for insectivorous bats also while feeding; (ii) surface water when drinking; (iii) cave roofs and walls as roost habitat; (iv) tree canopy for roosting or feeding. From these four habitats, potential transmission routes to infect bats from other hosts can be inferred (Table 1). In the following sections, the different transmission pathways that can link potential alternative hosts to bats are listed and discussed, along with examples of these alternative hosts.

## 4. Aerosol-Borne Route of Transmission

Firstly, EBOV transmission to bats could occur through aerosol transmission in all four habitats. This means that the maintenance host would release, in bats’ airspace, enough EBOV to contaminate bats. In theory, this would be possible in most bat environments, but we have discarded open-air transmission (e.g., in-flight bird to bat transmission) as the load of virus in the air cannot reach the levels that ensure infection. However, in the confined atmosphere of caves, bat to human transmission of rabies has been suspected [54,55,56]. EBOV and other filovirus particles seem to be able to persist for at least 90 min as aerosol [57,71], and experimental studies conducted on non-human primates (NHPs) by inoculating EBOV via the aerosol route were able to induce fatal disease 5 to 12 days post-inoculation [58]. Experimental airborne transmission of EBOV between animals from different species, e.g., from pigs to non-human primates, also seems possible [74]. In caves, the aerosol route might thus be possible. However, as bats tend to roost aggregated in groups and sometimes in large colonies, the ambient air may be saturated by bats’ aerosols, rather than an alternative host. Air screening could be attempted in bat habitats but experimental aerosol transmission trials from alternative hosts to bats would be more efficient.

## 5. Vector-Borne Route of Transmission

Bats are exposed to ectoparasitism [61]. If the biting invertebrate has previously bitten the alternative maintenance host, it could, in principle, infect bats. Hematophagous insects have been screened for EBOV during or after outbreaks with no conclusive results [26,75]. However, absence of exposure during an outbreak does not mean that the host is not involved in the maintenance of the virus in-between outbreaks. For example, the process of amplification in disease ecology can involve different hosts than maintenance hosts. Little information is available on ticks in bats. Ticks have been suggested to be involved in the transmission of Crimean-Congo haemorrhagic fever-like viruses to bats [76], and are seriously considered as potential hosts for the transmission of other pathogens from non-bat hosts to bats. Mosquitos could also be a vessel for a vector-borne transmission of EBOV. Studies on mosquito blood meals have revealed that mosquito could feed on bats and other mammals [62,63]. Bat flies appear to be highly bat-specific, adapted to their lifestyle [77,78,79,80] and are involved in the transmission of pathogens [64]. However, this specificity would preclude interspecies pathogen transmission. Ectoparasitism provides a potential solid source of indirect contacts between the alternative maintenance host and bats. This transmission pathway should be explored much further, and ecological insights, including insect and bat behavioural ecology, will be necessary to target the right insect species within the diversity of available biting species, in the right habitat (e.g., tree canopy level, caves’ roofs, when bats are immobile) at a proper time (e.g., nocturnal behaviour of bats) and season, when both hosts (i.e., the maintenance host and bats) can be fed upon by the vector. To our knowledge, such targeted protocols have not been implemented so far.

## 6. Insectivorous Food-Borne Route of Transmission (Insectivorous Bats)

Insectivorous bats feed on insects that could be a source of EBOV [61]. This food-borne route has been little investigated as well. A recent study pointed out the role of insect-specific viruses in the evolution of numerous viral families, including mononegaviruses, which infect vertebrates [81]. There is a possibility that prey-insects are the maintenance host for EBOV [61]. Insect vectors, such as blood feeding insects (e.g., mosquitos) could also, in theory, transport viruses in their blood meal after a bite on an infected host. They have been suspected in other filovirus outbreaks in the past [82]. In theory, these insects preyed upon by bats could also link bats to any type of maintenance host they could feed on. Bats actively search for prey in many different habitats hosting hematophagous insects that feed on habitat-specific fauna. Moreover, Reiskind et al. suggested that blood fed female mosquitos are more susceptible to predation [66]. Leendertz et al. also suggested that the population dynamics of mayflies may act as a driver of EBOV emergence in mammals and humans [46]. Insectivorous bat diet analysis could, therefore, indicate the relative proportion of hematophagous insect fed upon by bats and their identity, in order to subsequently target these insect species for sampling.

## 7. Environmental Route of Transmission

The EBOV maintenance host could shed viable viruses in the environment where bats could get infected by environmental exposure. The most likely habitats where this can happen are tree canopies and holes, and cave roofs/walls used only by a fraction of hosts inhabiting forests. The probability of infection will be dependent on the capacity of the virus to survive in the environmental conditions available in the specific habitat. Therefore, a better understanding of the capacity of EBOV to survive under different biotic and abiotic conditions is important to explore further (e.g., [71,73]). These experimental approaches should consider the specific environmental conditions occurring in the tree canopy and cave roofs in terms of substrate, temperature, humidity and light properties.

One particular mechanism that has been put forward in the literature is the fruit-borne route concerning frugivorous bats in the tree canopy. The availability of fruits attracts fruit-eating animals, including birds, tree-dwelling mammals, and invertebrates. This behaviour can create a network of contacts between hosts, leading to several transmission pathways, and this interaction network can be denser during seasons with food resource limitations [23,27]. Indirect contacts through faecal material, urine, or saliva left on fruits or branches could link the maintenance host with bats, in the same way that bats have been shown to be able to transmit other viruses (e.g., henipaviruses) through body fluids on fruit [33,70,83]. EBOV and filoviruses have been shown to persist for some time (3 to 7 days) in the environment, depending on the biotic and abiotic conditions [71,72,73]. In addition, EBV can be shed in some bat faeces [22] (but not all, [47]), and have been cultured from human urine and saliva [51], hence, could also be transmitted from faeces, urine, and saliva from other species. This transmission route is therefore possible, but restrained to the fauna feeding at the same height as bats (or, technically, above). The hypothesis of fruits soiled with infected body fluids falling on the ground and opening a transmission pathway towards other ground-level foraging hosts (e.g., duikers) does not expose bats to the alternative maintenance hosts (e.g., [83]).

## 8. Water-Borne Route of Transmission

A relation between river systems and EBOV outbreaks has been suggested in Central Africa, with tributaries influencing the spatial distribution of cases [84]. If river systems can harbour specific biotic communities with potential alternative hosts, such as water-dependent vectors [46], they can also represent, in remote forest ecosystems, the main transport pathways for people, providing a means for pathogens to spread through infected people or their hunted animals. Of course, in principle, while drinking, bats could get infected if the virus is present at the surface of the water. The capacity of EBOV to survive in the water has been the focus of a recent experimental study reporting an EBOV survival in water of 4 to 7 days between 21 and 27 °C [72]. Bats usually drink in open water, and not on the shores where viruses could be more concentrated by the presence of the maintenance host, for example. A dilution effect expected in open water, relative to some shallow water near the shores, would not favour such a transmission route a priori.

## 9. Direct Route of Transmission

Tree and cave roosts could expose hanging and resting bats to direct contact with a potential maintenance host. However, as a first observation, the upside-down vertical position of bat roosting does not really favour disease transmission from an alternative host. For bat species roosting in tree-holes, the situation can be different as they can share temporally or directly their nest space with other animals [85]. Secondly, the density of bats roosting in caves prevents the presence of many other potential hosts in the cave roof (but, for example, snakes can predate on bats in caves). During their feeding behaviour, frugivorous bats could be in direct contact with other hosts attracted by the fruits. Their nocturnal habits will limit the diversity of host they can interact with. We are not aware of any extensive study on the network of potential contacts between bats and other animals during their roosting and feeding behaviour. The majority of studies investigated potential of infectious contact from bats to other organisms [53]. Novel technologies, such as camera traps equipped with nocturnal vision, could provide opportunities for more research on this topic.

## 10. Other Animal-Borne Route of Transmission

As the ecology of most Africa bats is unknown, other opportunities exposing bat to potential maintenance hosts may be discovered in the future. For example, some bat species feed on fish [86] and, more recently, using stable isotopes of carbon and nitrogen as dietary tracers, it was demonstrated that a bat species, *Nyctalus lasiopterus*, was seasonally feeding on migrating Palearctic birds [87], a feeding behaviour unknown until now. Failed predation on bats could also be a rare opportunity for infectious transmission [52].

## 11. Research Perspectives

Considering the scenario B and C in Figure 1, that bats are not the maintenance hosts of EBOV or that they are not the only host involved in the maintenance of EBOV, helps in focusing EBOV research protocols on a reduced range of potential transmission routes and potential alternative hosts interacting with bats in their specific and limited habitats. This means that if bats are not the maintenance hosts for EBOV, then there is only a limited number of candidate species to play the role of alternative maintenance hosts. This limited number of alternative maintenance hosts is defined by the ecology of bats that imposes on those alternative maintenance hosts only a few possible EBOV transmission pathways towards bats. From the biodiversity of African forest and the full web of interactions between species, a set of secondary hypotheses indicated in Table 1 can be tested through protocols presented to further investigate the role of different maintenance host candidates for EBOV. The observation of this limited number of hosts calls for testing them, even if only to exclude them from the list of hypotheses and strengthen the main hypothesis. As warned above, the EBOV multi-host maintenance system could include a complex network of interacting bat species (Figure 1A2) and to proceed by elimination of alternative hypotheses may be a way to zoom-in on the maintenance community. The hypothesis of human playing a role in ebolavirus maintenance has not been addressed here, even if persistence of EBOV in previously infected humans has been recently proven [51]. This scenario would be more indicating of a change in the evolutionary trajectory of the pathogen (as moving from Step 4 to 5 in Figure 1 of Wolfe et al. [88]) than of the natural maintenance of ebolaviruses that is considered here.

In order for these protocols to be efficient and well designed, insights from behavioural ecology, plant phenology, and molecular biology (amongst other disciplines) will be necessary. Integrated approaches to health have been proposed recently and, in EBOV ecology, they should promote the integration of ecological sciences into health sciences that are usually at the forefront of epidemiological investigations. For example, a lot of sampling of potential alternative hosts has been undertaken during ebolaviruses outbreaks (e.g., [12,21,22,23,24,25,26]). These investigations concerned mainly the search for “what transmits ebolaviruses to people” as they were implemented during a human (or great ape) outbreak, and in the vicinity of outbreaks. This does not mean that they can automatically inform on “what maintains ebolaviruses”. When looking for the maintenance host, investigations should also target the same and other alternative hosts during inter-outbreak periods with ecologically driven hypotheses. This is what is currently done for bats following the main maintenance hypothesis (e.g., [30]), but not often for alternative hosts. Experimental trials should also concentrate on the environmental conditions occurring in bat-specific habitats, which can be very different from human outbreak conditions.

The transmission routes towards bats represent interhost contacts of unknown intensity and frequency, and it would be difficult to compare their relative importance. However, one can prioritize some transmission routes based on the current knowledge. The insect food-borne and vector-borne routes of transmission need, surely, to be further investigated, as they can expose bats to numerous other hosts. Previous works on insects have mainly concentrated on sampling insects in the human outbreaks’ surroundings (e.g., [26]). When searching for a maintenance host that can transmit EBOV to bats, protocols should concentrate on insects in interaction with known-exposed bat species. This would mean combining bat behavioural ecology and arthropod capture protocols to detect their potential carriage of EBOV, as well as protocols exploring bat feeding habits (e.g., molecular detection of prey DNA in bat’s guano) [65,67]. For example, insect captures should be targeted where insects can bite bats, in caves or at canopy level, and not at ground level where bats may not occur. Studying host interaction networks at fruit feeding sites is also an interesting avenue to explore direct, environmental, and fruit-borne routes of transmission. Behavioural ecology could inform and help targeting protocols. Chimpanzees and monkeys can feed at the same height as bats. Some rodent species feed on fruits, but the selection of the arboricolous species feeding at the same height as bats can reduce the list drastically. Camera trap protocols could inform host interaction networks placing bat species in symmetric or asymmetric interactions with other potential alternative hosts.

Under field reality, and especially in rainforests, this list of protocols will need a carefully designed programme to be successful, rooted in interdisciplinarity. As bats, and especially those species that have been exposed to ebolaviruses, are the entry point of most of these alternative hypotheses (i.e., alternative host need to be in contact with bats), the behavioural and community ecology of targeted bat species will need to be locally understood. Data recorders, such as vector or camera traps, will need to be deployed where bats are currently roosting or feeding. This can be a difficult task. Understanding which feeding resources attract bats at a specific season requires a good understanding of indigenous and domesticated tree phenology (e.g., [89]). Prior to this work, a guano-based dietary analysis of the feeding behaviour of bats could help to map locally where and when bats will be present. Then, simultaneous protocols on bats and sympatric alternative hosts can be implemented, and a biological search for antibodies or antigens can be implemented. Combining protocols to test the main and alternative hypotheses could provide cost-effective and synergetic options.

To conclude, alternative hypotheses presented here should be explored alongside efforts to confirm bat species as maintenance hosts for EBOV. The ecology of those bat species already known to be exposed should be used to design protocols in order to target relevant alternative maintenance hosts. Given the number of species already involved/exposed to EBOV, the ecology of EBOV and its maintenance system can be expected to be complex, ecosystem dependent [46], and dynamic, due to global changes [90]. The Ebola maintenance system, once isolated in the forests, is now interacting with humans and their modified environments and will adapt to it. Aiming at this moving target will require out-of-the-box thinking and interdisciplinary collaboration.

## Figures and Tables

**Table 1:** Hypothetical transmission pathways between the maintenance host of EBOV and bat hosts under *H0* as described in the main text, classified by habitat used by bats, and field and experimental protocols to test them.

Transmission Pathways	Habitat	Bat Behaviour	Research Protocols	Existing Literature/ Methods
Air-borne	All	All	- Experimental EBOV interspecies aerosol transmission under rainforest conditions - Experimental EBOV excretion study in potential maintenance hosts - Experimental EBOV droplet survival in specific habitat conditions	[22,44,45, 54,55,56,57,58,59,60]
Vector-borne	Tree-canopy Cave roof	Feeding Roosting	- Vector feeding habit (blood meal) to identify vector feeding host range, including bats - EBOV screening in vector populations	[61,62,63,64,65]
Food-borne (insects)	Open Air Surface water	Feeding Drinking	- Genetic screening of prey species in bat guano- EBOV screening in vector populations - Vector feeding habit (blood meal) to identify vector feeding host range	[46,62,63, 65,66,67,68,69]
Food-borne (fruits)	Tree-canopy	Feeding	- Experimental EBOV environmental/fruit survival in specific habitat conditions - Behavioural study (e.g., camera trap) in canopy habitat to identify social network between bats and other potential hosts - EBOV screening of frugivorous vertebrates (feeding in tree canopy)	[70]
Water-borne	Surface water	Drinking	- Water screening for EBOV (open vs shallow waters) - Experimental EBOV water survival under rainforest conditions	[46,71,72]
Direct	Tree-canopy Cave roof	Feeding Roosting	- Behavioural study (e.g., camera trap) in canopy and cave habitat to identify social network between bats and other potential hosts - EBOV screening of frugivorous and arboricolous vertebrates and invertebrates - EBOV screening of cave roof dwelling animals	[26]
Environmental	Tree-canopy Cave roof	Feeding Roosting	- Experimental EBOV environmental survival on different substrates and environmental conditions specific to bat habitats	[71,73]
